# Extraocular silicone oil deposition: a clinicopathologic case series

**DOI:** 10.1007/s00417-026-07229-w

**Published:** 2026-04-11

**Authors:** Nicholas Joseph DeLuca, Matthew Camacho, Maria Idarraga, Sander R. Dubovy

**Affiliations:** 1 The Florida Lions Ocular Pathology Laboratory, Bascom Palmer Eye Institute, University of Miami Miller School of Medicine, Florida Lions Eye Bank, 900 NW 17th St., Miami, FL 33136 USA; 2 The University of Tennessee Health Science Center, Memphis, TN USA

**Keywords:** Conjunctiva, Extraocular migration, Eyelid, Histopathology, Immunohistochemistry, Intraocular tamponade, Orbit, Silicone oil, Vitrectomy, Vitreous substitute

## Abstract

**Purpose:**

To characterize the clinicopathologic features of extraocular silicone oil (SO) migration in the ocular adnexae, including the conjunctiva, eyelid, and orbit (CEO).

**Methods:**

Retrospective, single-institution case series. The Florida Lions Ocular Pathology Laboratory database at the Bascom Palmer Eye Institute was searched for surgical specimens diagnosed with “silicone oil” involving the ocular adnexae from 1997 to 2023. Clinical and histopathologic features were reviewed. All specimens were stained with hematoxylin-eosin, and immunohistochemical stains for histiocytes CD68 (M1, pro-inflammatory) and CD163 (M2, anti-inflammatory).

**Results:**

25 specimens met inclusion criteria. Affected sites included the conjunctiva (76%), orbit (12%), eyelid (8%), and Tenon’s capsule (4%). Histopathology revealed clear dropout spaces consistent with SO surrounded by histiocytes in all cases. CD68 and CD163 both highlighted histiocytes, with CD163 demonstrating significantly higher immunohistochemical staining (*p* < 0.0001). The average time from SO placement to presentation was 451.1 days (Range: 14 − 1,220 days). SO migration was clinically suspected in 17 cases, presenting with conjunctival injection, irritation, cysts, or eyelid swelling. Imaging (anterior segment OCT, CT) aided diagnosis.

**Conclusion:**

Diagnosis of silicone oil migration in the ocular adnexae is important to avoid misdiagnosis. The presence of M2 histiocytes (anti-inflammatory) is consistent with the high immunotolerance of these lesions.

## Key messages

*****What is known***:**

- Extraocular migration of intraocular silicone oil is an uncommon but recognized complication after vitreoretinal surgery and can present as conjunctival, eyelid, or orbital lesions.

*****What is new***:**

- This is the largest clinicopathologic case series to date of extraocular silicone oil deposition involving the conjunctiva, eyelid, and orbit.
- Lesions show a predominance of CD163-positive (M2) histiocytes, suggesting an immunotolerant/anti-inflammatory response to extravasated silicone oil.
- AS-OCT and CT can help identify and localize extraocular silicone oil prior to surgical excision.

## Introduction

Silicone oil (SO), introduced in 1962 as a vitreous substitute in the treatment of retinal detachment [1], remains a common long-term tamponade used after vitrectomy for indications including rhegmatogenous retinal detachment, proliferative vitreoretinopathy, ocular trauma, endophthalmitis, chronic hypotony, and chronic retinoschisis. The chemical properties of SO allow for similar refractive index, longevity, and stability when compared to vitreous [2, 3]. Additionally, the high interface tension with water prohibits the migration of aqueous humor into the subretinal space [3]. SO may be used with great efficacy in children, intellectually disabled adults, patients whose occupation requires high altitude travel, and patients for whom postoperative positioning (e.g., face-down positioning) is not feasible [2, 3].

The efficacy of SO is limited by lipophilic emulsification and underpins the recommendation for removal once stabilization of the primary process is achieved. Known complications include cataract, SO-related visual loss, secondary glaucoma, keratopathy, and subretinal and extraocular migration [4]. The continued use and potential as a drug-eluting agent elevates the importance of understanding complications including extraocular migration [2, 3]. Novel replacements for vitreous humor including the foldable capsular vitreous body, hydrogel-based intravitreal tamponade, and medium-chain triglycerides are being developed to mitigate the aforementioned complications of SO [3].

Central nervous system (CNS) migration of the oil into the intracerebral lateral ventricles and intracranial optic nerve have been reported [5–7]. Cavernous degeneration of the optic nerve via increased intraocular pressure has been a proposed mechanism for migration into the CNS (similar to Schnabel’s cavernous optic nerve atrophy) [8]. Other theorized mechanisms include aberrant connections between the vitreous space and subarachnoid space through congenital abnormalities and migration of silicone-laden macrophages [6].

Vitreoretinal surgeons must be aware of the potential for SO migration in globes with normal to high intraocular pressure through places of potential egress created by surgery, including sclerostomies, perforation sites, suture tracks, glaucoma drainage devices, and during removal [4, 9]. Recent reviews have found that orbital migration was associated with a history of trauma, aphakia, glaucoma drainage devices, and scleral buckle [4, 10]. Lam et al. found that orbital migration of SO often required surgical intervention with 94.1% of eyes in their sample requiring surgical intervention, most often for ptosis [4]. Clinically, SO migration in the eyelids may present as pseudo-xanthelasma, ptosis, and emphysema [9, 11, 12].

SO, when extravasated, induces chronic granulomatous inflammation and may form silicone vacuoles that create cavernous spaces within adjacent tissues, irrespective of the oil’s viscosity. These vacuoles are typically surrounded by oil-laden macrophages [4, 9, 13]. Histopathologic examination reveals histiocytes encircling clear dropout spaces. The immunophenotypic characterization of these histiocytes remains incompletely understood. Broadly, histiocytes can be categorized into two subtypes: M1 and M2. M1 histiocytes are associated with pro-inflammatory response, including phagocytosis and recruitment of additional immune cells, while M2 histiocytes mediate anti-inflammatory effects through mechanisms including phagocytosis, fibrosis, and tissue repair [14]. The inflammatory milieu associated with SO migration is predominantly histiocytic, with minimal infiltration by neutrophils, lymphocytes, or plasma cells. Immunohistochemical staining for CD68 and CD163 can be used to identify M1 and M2 histiocytes, respectively [14].

This case series characterizes the clinicohistopathologic features of extravasated silicone oil (SO) in the ocular adnexae, including the conjunctiva, orbit, and eyelid. These findings aim to enhance understanding of this rare complication (incidence of 3–30% migration to subconjunctival space [4]) associated with a commonly used endotamponade agent and contribute to the growing literature on extraocular SO migration. To our knowledge, this represents the largest case series on the clinicopathologic correlation of extraocular SO. Specifically, the study describes the clinical presentation and histopathologic morphology with characterization of the inflammatory infiltrate associated with these lesions.

## Methods

This is a retrospective, single institution, case series. The database at the Florida Lions Ocular Pathology Laboratory of the Bascom Palmer Eye Institute was searched for surgical specimens with a diagnosis of “silicone oil” in the conjunctiva, orbit, or eyelid on histopathology between the years of 1997 and 2023 from the Florida Lions Ocular Pathology laboratory database. 25 surgical specimens were included. Patient demographics (age and gender), anatomic site, laterality, clinical features, and ocular examination findings were obtained from available medical records. All specimens were stained with hematoxylin and eosin, CD68 IHC (Leica Biosystems, Germany), and CD163 (Leica Biosystems, Germany). Immunohistochemical staining intensity for CD68 and CD163 were graded qualitatively on an ordinal scale from 0 to 3 by a board-certified anatomic pathologist at the Florida Lions Ocular Pathology Laboratory. The staining scores for the two markers were compared using a paired *t*-test which was performed in Microsoft Excel to evaluate differences in macrophage subtype predominance. Interquartile ranges were calculated using the 25th and 75th percentiles.

## Results

**Fig. 1:**
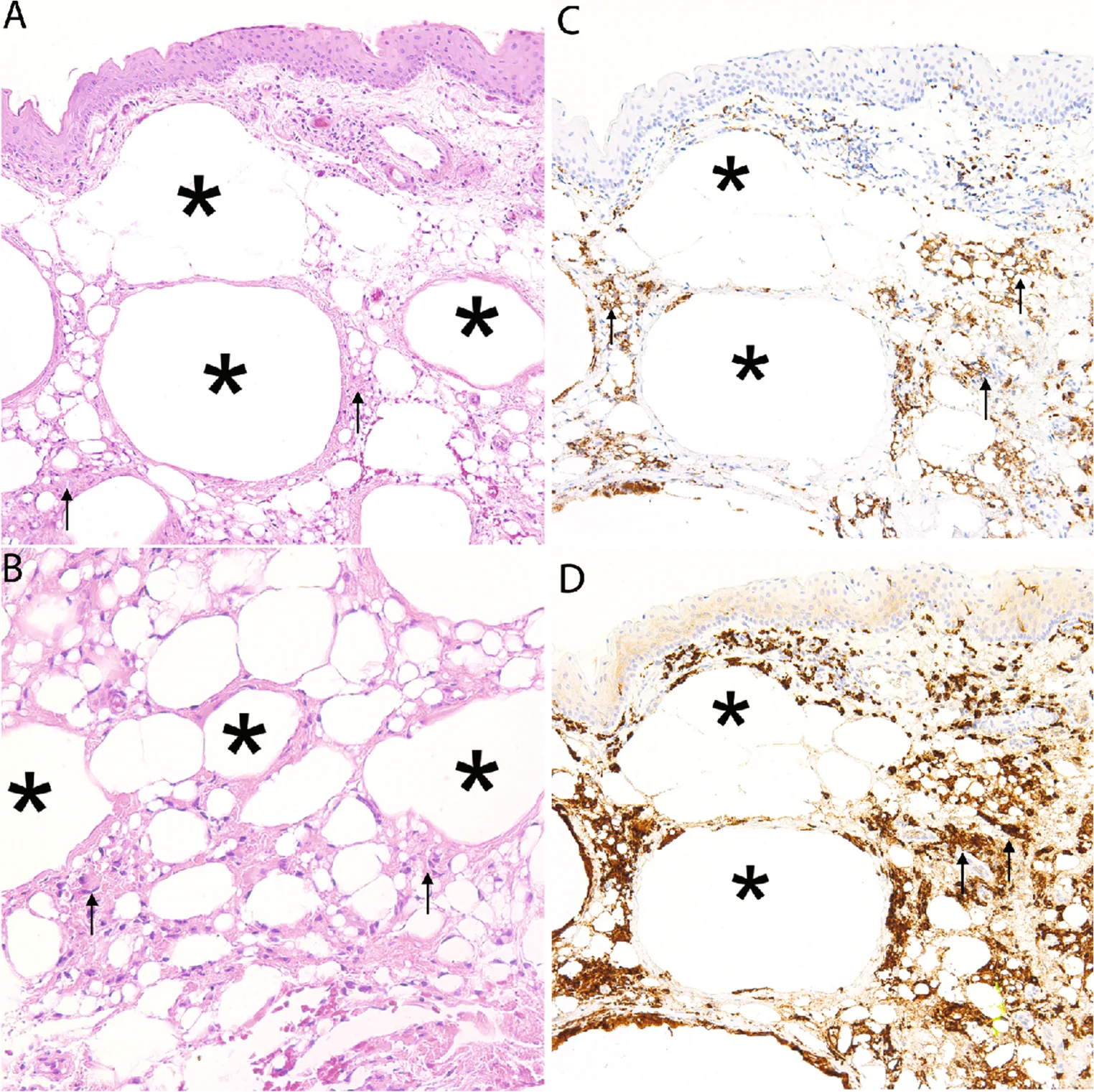
Histopathologic and immunohistochemical features of silicone oil in the conjunctiva. (**a**) Clear dropout spaces (silicone oil, asterisks) surrounded by histiocytes and fibrous tissue (arrows) within the substantia propria (Hematoxylin-eosin, original magnification x 200). (**b**) High power demonstrates clear spaces (silicone oil, asterisks) of variable size with surrounding histiocytes and fibrous tissue (arrows) (Hematoxylin-eosin, original magnification x 400). (**c**) CD68 (pro-inflammatory) IHC highlights the histiocytes present within the substantia propria (arrows) surrounding the clear spaces (silicone oil, asterisks) (CD68, original magnification x 200). (**d**) CD163 (anti-inflammatory) IHC highlights the histiocytes present within the substantia propria (arrows) surrounding the clear spaces (silicone oil, asterisks) (CD163, original magnification x 200)

**Fig. 2:**
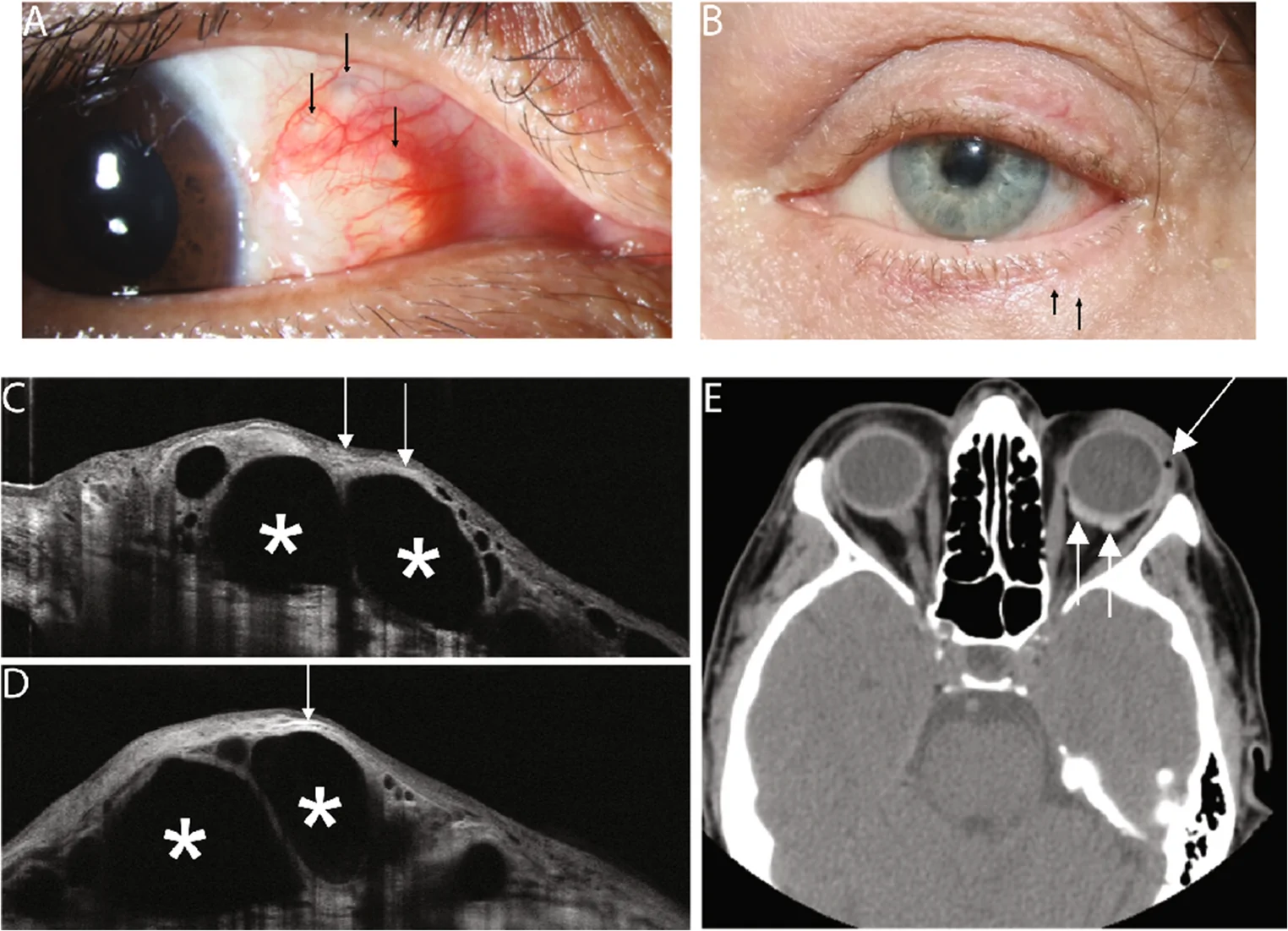
**R**epresentative images of silicone oil lesions in the bulbar conjunctiva and eyelid, and silicone oil lesions on anterior segment OCT and CT imaging of the brain and orbits. (**a**) Cystic lesion consisting of silicone oil present in the right nasal bulbar conjunctiva (arrows). (**b**) Extravasated silicone oil presents as a palpable lesion with fullness in the left lower eyelid (arrows). (**c**,** d**) Anterior segment OCT demonstrates an unremarkable hyporeflective epithelium with subjacent variably hyperreflective substantia propria (arrows). Underlying hyporeflective cystic spaces (asterisks) corresponding to silicone oil surrounded by histiocytes. (**e**) CT scan of the brain and orbits demonstrates thickening in a retrobulbar and lateral conjunctival location (arrows) corresponding to silicone oil migration with surrounding histiocytic infiltrate

The diagnosis of extraocular SO deposition was established by light microscopic examination of formalin fixed, paraffin embedded tissue in 25 patients. There were 10 right eyes and 15 left eyes. Of the 25 cases, there were 19 males and 6 females. Mean age was 38.9 years (Range: 8–65 years). Anatomic sites affected included the conjunctiva (19; 76%), orbit (3; 12%), eyelid (2; 8%), and Tenon’s capsule (1; 4%, included in conjunctiva). Combined involvement of multiple periocular structures was identified in one case, with silicone oil present in both the orbit and eyelid. All 25 cases demonstrated histiocytes surrounding clear dropout spaces containing presumed SO (Fig. 1a and b). CD68 and CD163 immunohistochemistry highlighted the aforementioned histiocytes in all cases (Fig. 1c and d). The descriptive statistics for the grading of CD68 and CD163 as well as their ratio can be found in Table 1. The mean grade for CD163 (x̄ = 2.38) was significantly higher than that for CD68 (x̄ = 1.56, *p* < 0.0001). The average time from SO placement to extraocular presentation was 451.1 days (16 cases; Range: 14 − 1,220 days). SO migration was clinically suspected in 17 cases (68%). Clinical presentations in conjunctival lesions (of 9 available records) included conjunctival injection (6), irritation/discomfort (5), and asymptomatic cyst(s) (4). Presentation in the eyelid included palpable lower eyelid swelling without erythema or pain in one case (Fig. 2e). No clinical history was available in the orbital case. Clinical indications for SO are presented in Table 2. Coherence tomography (CT) (*n* = 1) and anterior segment optical coherence tomography (AS-OCT) (*n* = 2) aided in identifying the extravasated material.

**Table 1:** CD68 vs. CD163 Immunohistochemical Grading

Descriptive Statistics	CD68 (*n* = 25)	CD163 (*n* = 25)	CD68/CD163 Ratio (*n* = 25)
Mean ± SD	1.56 ± 0.63	2.38 ± 0.78	0.66 ± 0.18
Standard Error	0.13	0.16	0.04
Minimum	0.5	1	0.25
Maximum	3	3	1
Median	2	3	0.67
Interquartile Range (IQR)	1	1	0.17

**Table 2:** Primary etiology requiring silicone oil placement

Clinical entity	Cases (%)
Retinal detachment (not specified)	7 (28)
Ocular trauma	6 (24)
Proliferative diabetic retinopathy*	4 (16)
Proliferative vitreoretinopathy*	2 (8)
Malignancy	1 (4)
Endophthalmitis	1 (4)
Other^†^	4 (16)

*Includes cases with and without retinal detachment

^†^Coat’s disease (1), Myopia with RD, X-linked retinoschisis with RD, No history (1)

## Discussion

Silicone oil has been widely used as a tamponade agent and vitreous substitute in various vitreoretinal diseases with many known complications resulting from lipoemulsification (e.g., early cataract formation, keratopathy, glaucoma, and retinal toxicity) [2, 3]. Despite this, SO remains a mainstay treatment of various retinal pathologies.

Extraocular migration of silicone oil is thought to occur through surgically created pathways following vitreoretinal surgery, including sclerotomy sites, perforation sites, suture tracks, or glaucoma drainage devices, allowing emulsified oil droplets to track along periocular tissue planes. Silicone oil migration into the ocular adnexa is well documented and presents in varied ways [4]. It commonly involves the conjunctiva in areas surrounding sclerotomies. In our cases, the most common presenting signs were a painless, clear cystic lesion with localized injection (Fig. 2d). Clinical suspicion for SO involvement remained high in these cases and simple excision was curative. However, in orbital or eyelid involvement, the presentation was nonspecific with painless swelling and may raise concern for malignancy (Fig. 2e). In the current study, SO migration was often suspected when retinal surgery was recent and/or presented in the conjunctiva. Surgical removal of the adnexal lesion is generally recommended to confirm the pathology and rule out malignancy. The time of presentation following retinal surgery varied greatly in our study (14 − 1,220 days). Review of the available clinical charts did not identify a consistent pattern of postoperative complications associated with the development of extraocular silicone oil migration in this series. None of the eyes in this series demonstrated phthisical changes at the time of presentation with extraocular silicone oil deposition.

Imaging modalities such as AS-OCT and CT of the brain and orbits can be utilized to characterize the lesions. AS-OCT is most useful in bulbar conjunctival lesions and may demonstrate unremarkable hyporeflective epithelium with subjacent variably hyperreflective substantia propria overlying hyporeflective cystic spaces corresponding to silicone oil surrounded by histiocytes (Fig. 2a and b). CT of the brain and orbits may be useful in characterizing the spatial orientation of eyelid and orbital lesions prior to surgery (Fig. 2c). CT can also aid in ruling out brain and optic nerve involvement. In our series, CT imaging demonstrated retrobulbar and lateral conjunctival soft tissue thickening corresponding to extraocular silicone oil migration.

Histopathology demonstrated enlarged clear cystic spaces lined by histiocytes indicating a foreign body reaction (Fig. 1a and b). There was a paucity of lymphocytes, plasma cells, and neutrophils in the available sections. The cystic spaces were large and lined by CD68 and CD163 positive histiocytes (Fig. 1c and d). Immunohistochemistry with CD68 and CD163 revealed a dual population of M1 and M2 histiocytes, respectively (Fig. 1). CD163-positive histiocytes were significantly more abundant than CD68-positive histiocytes, indicating a predominance of the M2 phenotype. This immunophenotype is consistent with a reparative immune response, as M2 histiocytes are known to mediate phagocytosis, fibrosis, and tissue remodeling. Given the known clinical tolerance of SO lesions, the predominance of M2 histiocytes supports the notion of an adaptive, anti-inflammatory environment [14]. The presence of both pro-inflammatory and anti-inflammatory histiocytes reflects the complex immunologic dynamics of the foreign body reaction. The histomorphologic appearance of these lesions may mimic lipomas, liposarcomas, and epithelioid hemangioepitheliomas, thus, it is important for pathologists to recognize SO migration from atypical lipomatous lesions.

This study was limited in its ability to evaluate the impact of surgeon experience on SO deposition. Although the operating surgeon could be identified, the degree of trainee involvement and the surgeon’s level of experience at the time of surgery could not be reliably determined from the available records. Visual acuity outcomes were also not systematically analyzed in this pathology-based series. Although the time interval between silicone oil placement and extraocular presentation was recorded, it was not always possible to determine whether changes in vision were temporally related to the development of extraocular silicone oil migration. When visual acuity data with clearly documented temporality were available, vision was generally preserved in cases with orbital involvement. In addition, recurrence following excision was not specifically evaluated in this series.

## Conclusion

This case series of 25 patients represents the largest single-institution analysis of extraocular SO migration with histopathologic correlation. Although rare, SO migration can involve various periocular structures, leading to chronic inflammation, mass effect while mimicking neoplastic processes. This often presents months to years post-vitrectomy. Recognition of this complication is important for ophthalmologists and pathologists as most cases may be resolved by simple surgical excision. Imaging modalities including anterior segment OCT and CT can assist in identifying extravasated material. While it remains unclear whether low- or high-viscosity SO is more prone to migration, physical properties alone would suggest lower viscosity may pose greater risk.

## Data Availability

The dataset used in this study was derived from the Florida Lions Ocular Pathology Laboratory database and associated medical records. These data are not publicly available due to HIPAA protections and institutional policies. De-identified data may be shared upon reasonable request and subject to applicable institutional approvals.
